# Assessing the fidelity of a behavioural intervention involving academic detailing in general practice: a sub-study of the ‘Implementing work-related Mental health guidelines in general PRacticE’ (IMPRovE) trial

**DOI:** 10.1186/s43058-023-00531-2

**Published:** 2023-11-29

**Authors:** Vera Camões-Costa, Samantha Chakraborty, Fatima Mozaffari, Alexander Collie, Justin Kenardy, Bianca Brijnath, Duncan Mortimer, Joanne Enticott, Michael Kidd, Lyndal Trevena, Sharon Reid, Danielle Mazza

**Affiliations:** 1 Department of General Practice, School of Public Health and Preventive Medicine, Monash University, Melbourne, Australia; 2 Healthy Working Lives Research Group, School of Public Health and Preventive Medicine, Melbourne, Australia; 3 University of Queensland, Brisbane, Australia; 4 National Ageing Research Institute, Parkville, Australia; 5 Centre for Health Economics, Monash Business School, Monash University, Melbourne, Australia; 6 Monash Centre for Health Research and Implementation, Monash University, Melbourne, Australia; 7 College of Health and Medicine, Australian National University, Canberra, Australia; 8 Australian Government Department of Health and Aged Care, Canberra, Australia; 9 Faculty of Medicine and Health, School of Public Health, The University of Sydney, Camperdown, Australia; 10 Specialty of Addiction Medicine, Central Clinical School, Faculty of Medicine and Health, The University of Sydney, Camperdown, Australia

**Keywords:** Implementation fidelity, General practice, Assessment, Academic detailing, Conceptual framework

## Abstract

**Background:**

Assessing the fidelity of intervention components enables researchers to make informed judgements about the influence of those components on the observed outcome. The ‘Implementing work-related Mental health guidelines in general PRacticE’ (IMPRovE) trial is a hybrid III trial aiming to increase adherence to the ‘Clinical Guidelines for the diagnosis and management of work-related mental health conditions in general practice’. IMPRovE is a multifaceted intervention, with one of the central components being academic detailing (AD). This study describes the fidelity to the protocol for the AD component of the IMPRovE intervention.

**Method:**

All AD sessions for the trial were audio-recorded and a sample of 22% were randomly selected for fidelity assessment. Fidelity was assessed using a tailored proforma based on the Modified Conceptual Framework for fidelity assessment, measuring duration, coverage, frequency and content. A descriptive analysis was used to quantify fidelity to the protocol and a content analysis was used to elucidate qualitative aspects of fidelity.

**Results:**

A total of eight AD sessions were included in the fidelity assessment. The average fidelity score was 89.2%, ranging from 80 to 100% across the eight sessions. The sessions were on average 47 min long and addressed all of the ten chapters in the guideline. Of the guideline chapters, 9 were frequently discussed. The least frequently discussed chapter related to management of comorbid conditions. Most general practitioner (GP) participants used the AD sessions to discuss challenges with managing secondary mental conditions. In line with the protocol, opinion leaders who delivered the AD sessions largely offered evidence-based strategies aligning with the clinical guideline recommendations.

**Conclusions/implications:**

The IMPRovE AD intervention component was delivered to high fidelity. The sessions adhered to the intended duration, coverage, frequency, and content allowing participating GPs to comprehend the implementation of the guideline in their own practice. This study also demonstrates that the Modified Conceptual Fidelity Framework with a mixed methods approach can support the assessment of implementation fidelity of a behavioural intervention in general practice. The findings enhance the trustworthiness of reported outcomes from IMPRovE and show that assessing fidelity is amenable for AD and should be incorporated in other studies using AD.

**Trial registration:**

Australian New Zealand Clinical Trials Registry ACTRN 12620001163998, November 2020.

**Supplementary Information:**

The online version contains supplementary material available at 10.1186/s43058-023-00531-2.

Contributions to the literature

- This study demonstrates the value of utilising mixed methods to assess fidelity of a complex behavioural intervention.
- This study identifies the strengths (and limitations) of the Modified Conceptual Framework for Implementation Fidelity as a valid and comprehensive method to assessing fidelity.
- This study supports the use of this methodology in future studies that assess the implementation fidelity of complex behavioural interventions in general practice.

## Introduction

Behavioural interventions aim to facilitate change in a participant’s behaviour with potential for improving health outcomes [1]. These types of interventions are often multifaceted. However, determining the contribution of each component [2] of a multifaceted behavioural intervention to the outcome can be challenging [2]. This is because the observed outcome may be influenced by each individual components, the impact of one component on another, or the impact of real-world contexts where an intervention is being delivered [3]. The influence of real-world contexts is greater for behavioural interventions, compared with non-behavioural interventions, as differences in how practitioners deliver practitioner-led components the extent to which participants respond to the intervention, or the broader social context, can all alter the delivery of the intervention and therefore its fidelity throughout a trial [4–6]. An intervention delivered with high fidelity provides healthcare decision-makers and researchers with greater confidence in results that might otherwise come from undefined confounding variables [7]. Understanding the fidelity of an intervention enables researchers to comprehend how and why its specific elements may or may not work [8] thus highlighting implications for translation to larger-scale community settings.

There is currently limited consensus on the key elements involved in studying intervention fidelity in randomised controlled trials, including trials of multifaceted behavioural interventions [3, 7–9]. For multifaceted interventions, it is recommended that fidelity should be assessed as the ‘function and process’ of the intervention, rather than the intervention components themselves. For behavioural interventions however, it is important to also assess adherence to the protocol, dose of the intervention delivered, quality of programme delivery, participant responsiveness and programme differentiation. These aspects can be assessed individually for each component and subsequently nested in a larger assessment of function and process [7, 10]. Assessing both the fidelity of individual components and combined fidelity of a multifaceted intervention would provide a comprehensive assessment of the fidelity of multifaceted behavioural interventions.

The IMPRovE trial aimed to assess the effectiveness of a multifaceted intervention on the implementation of the ‘Clinical guideline for the diagnosis and management of work-related mental health conditions in general practice’. It investigated the effectiveness of a multifaceted intervention, comprising academic detailing (AD), enrolment in a digital community of practice and the provision of resources, on guideline adherence. [11]. The first and key element of the IMPRovE intervention, AD, is increasingly used to change practitioners’ behaviour and enhance evidence-based practice. It is hypothesised to effect behaviour through opinion leader-led and personalised discussion and education. In general practice, AD involves a visit from a trained health care professional to provide evidence-based education on a chosen topic. It used commonly to optimise prescribing [12, 13] but is also used to increase evidence-based care for topics such as management of breathlessness in people with advance stage cancer [12, 14]. In the context of the IMPRovE trial, AD consisted of a single 60-min meeting that was co-delivered by a GP opinion leader and an experienced academic detailer via video conferencing, whereby participating GPs analysed case studies comparing patient care against published mental health guidelines, evaluated their own current practice against work-related mental health guidelines, and discussed strategies to diagnose and manage work-related mental health conditions according to evidence-based recommendations. Additional File 1 describes the plan for AD sessions within the IMPRovE trial.

In this paper, we describe the extent to which the AD component of the IMPRovE trial, was delivered according to its pre-defined process and intended function [11].

## Method

**Fig. 1:**
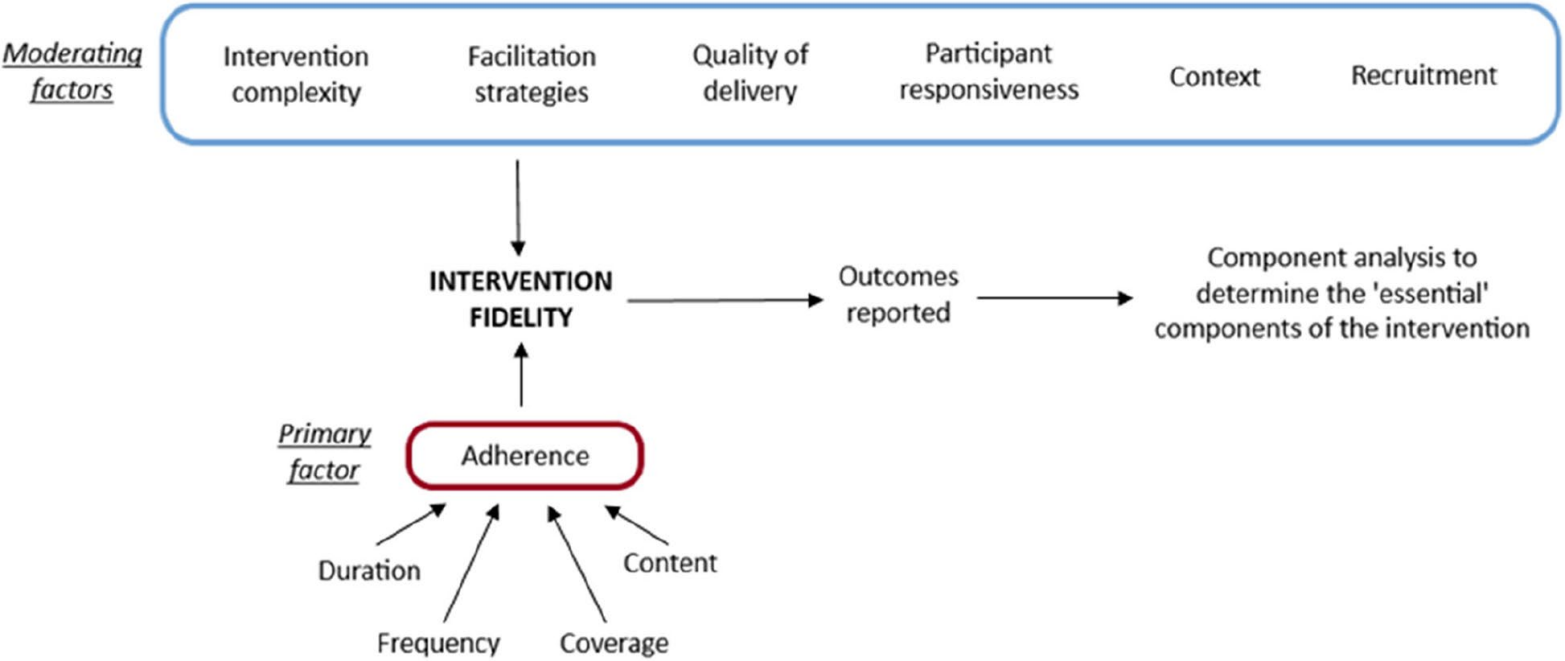
Modified Conceptual Framework for implementation fidelity

We utilised the Conceptual Framework for Implementation Fidelity, as proposed by Carroll et al. [7] and modified by Hasson [10] (see Fig. 1), to guide how we assessed the fidelity of the AD component of our intervention and the rationale for focusing the fidelity assessment on adherence to the intervention. The framework suggests the primary factor affecting intervention fidelity is adherence, but the broader implementation process (and its fidelity) may be also affected by different moderating factors, including intervention complexity, facilitation strategies, quality of delivery, participant responsiveness, context, and recruitment. Facilitation strategies, such as the provision of delivery manuals and training, may lead to better implementation fidelity compared to interventions with less delivery guidance. The appropriateness of the delivery process for achieving what was intended, as well as the enthusiasm of those delivering and receiving the intervention, may also affect fidelity-related outcomes. In addition, the use of delivery guidance may improve quality of the delivery, which in turn may influence participants’ uptake of the intervention. Finally, contextual influences, such as system structure or culture, and aspects related to recruitment, such as what motivated participants’ involvement or the value they see in the intervention, can influence fidelity. Moderators such as complexity engaging with other components of the IMPRovE intervention, receipt or accessing of manuals and training materials, and enthusiasm of those involved in the intervention, are likely to influence adherence to the intervention and subsequently outcomes of the IMPRovE trial. Given the potentially wider impact of moderating factors on the trial outcomes, these factors will be reported in the trial realist evaluation (publication pending).

Measurement of adherence to the IMPRovE AD plan includes duration, frequency, coverage and content, as outlined in Table 1.

**Table 1:** Elements of adherence measured in the IMPRovE AD plan

Adherence	The extent an intervention is delivered as intended according to intervention protocol
Duration	The duration of the delivered intervention
Content	The intervention’s ‘active ingredients’: encompassing the knowledge and skills to be delivered in those intervention sessions
Frequency	The frequency with which the intervention’s active ingredients were delivered during the intervention
Coverage	A measure of how much of the intervention’s prescribed content was delivered

All AD sessions that were delivered within the IMPRovE intervention were audio-recorded. A minimum of 20% of the AD sessions delivered by each opinion leader were chosen for assessment, with 22.8% of all the delivered AD sessions eventually selected. This sample of AD sessions was selected through stratified random sampling, stratified by opinion leader (in order to not bias the sample to any particular opinion leaders’ adherence and to adequately represent each opinion leader who delivered the AD session) and by state. Randomisation was conducted by an independent statistician after all the AD sessions were delivered.

We used a mixed method triangulation design, giving equal weight to both quantitative and qualitative data collected and analysed to provide an integrated understanding of the breadth and depth of adherence to the planned design of the AD sessions [15–17]. A proforma was developed to record fidelity elements prior to commencing delivery of AD sessions (Additional File 2). To collect data for the fidelity assessment, two assessors (FM or CR and KN) listened to recordings of each included AD session and completed a proforma describing the presence of facilitators (academic detailers and opinion leaders) and GP participants in the trial, the duration of the sessions, the frequency with which the opinion leaders referred to the guidelines, the challenges described and the case studies utilised (i.e. coverage and content) [18].

Quantitative scoring criteria for the fidelity assessment were based on the adherence component of an adapted model of the Modified Conceptual Framework for Implementation Fidelity [7, 10], which involved an assessment of the duration, content, frequency and coverage of the AD sessions, as outlined in Table 2. A point was awarded for each item addressed in the criteria, comprising 1 point for duration, 7 points for content, 3 points for frequency and 4 points for coverage, giving a maximum total score of 15. Whilst there was no pre-determined frequency of references to the guideline that could be defined as ‘optimal’, increased frequency was anticipated to provide GPs with a more comprehensive understanding of the guideline, and therefore higher fidelity.

**Table 2:** Criteria used for the fidelity analysis of the AD sessions of the IMPRovE trial adapted from Carroll et. al [7]

Section	Fidelity criterion
Duration	The session duration was within 50 min ± 10 min
Content	*The opinion leader/academic detailer*:
	Introduced the session by describing the importance of GPs in patient recovery
	Incorporated challenges from the participating GP in the AD session
	*The participating GPs*:
	Evaluated their own practice against the work-related mental health guidelines
	Created strategies to diagnose and manage work-related mental health conditions according to evidence-based recommendations
	*Highlighted the 3 key messages of the session*:
	Treat the person in their situation
	Offer case that is within your scope of practice and use your network to provide collaborative care
	Good work is good for recovery
Frequency	*The guideline recommendations were referred to*:
	5–9 times (1 point)
	10–14 times (2 points)
	15–20 times (3 points)
Coverage	The GP opinion leader (OP) and the Academic detailer were present in the sessions
	All consenting GPs from the practice attended the session
	*The opinion leader/academic detailer*:
	Hosted a discussion about the challenges in practice
	Used a case study to facilitate the discussion

Academic detailers were employees of the National Prescribing Service (NPS) MedicineWise, an Australian not-for-profit organisation with extensive experience in delivering educational outreach sessions to GPs on therapeutics. Their role during the AD session was to run the session and help facilitate discussion. GP opinion leaders were clinicians well-versed in compensation schemes. They were nominated by project partners, thus demonstrating their leadership amongst their peers. Opinion leaders provided a peer’s voice during the session, facilitating greater engagement from participating GPs [19]. They had extensive knowledge of the guideline recommendations and experience in their application in practice. A total of two academic detailers and six GP opinion leaders were employed to deliver the AD to the 38 clusters of GPs in the intervention arm of the trial [11]. The assessment of coverage involved: the presence of both facilitators (the GP opinion leader and the academic detailer), whether all participating GPs from the practice attended the sessions, whether there was a discussion on challenges in the practice, and whether a case study was discussed (supplied in Additional File 3).

Qualitative data were analysed using a conventional content analysis approach [18] which is appropriate because it provides a descriptive account of the adherence to the AD protocol. First each assessor (FM or CR and KN) listened to the recorded sessions, then independently listed and categorised the challenges that arose from the recorded discussions. These challenges were categorised according to the 10 clinical questions that address the known GP challenges that make up the 10 chapters of the Guideline. To establish the reliability of the coding undertaken within the session and to limit subjectivity, each AD session was coded by two researchers independently, and a third researcher was involved in coding when diverging coding needed to be resolved and until consensus was achieved. These researchers were not in intervention development or delivery.

All AD sessions were scored using the fidelity coding assessment sheet (Additional File 2), and both quantitative and qualitative fidelity assessment was undertaken in Microsoft Office Excel (V.2111) spreadsheets.

## Results

Fidelity assessment was undertaken on a stratified random sample of 8 AD sessions from a total of 35 AD sessions. These sessions were delivered to a total of eight participating GPs across all states and territories in Australia. A total of five opinion leader GPs and two academic detailers delivered the intervention for the sample.

For the sample of AD sessions assessed, the observed adherence to the planned design of the AD sessions averaged 89.2%, ranging from a minimum score of 80.0% to a maximum of 100.0%. The fidelity of each AD session, across the four elements of adherence, and the various challenges raised by GPs in the eight AD sessions as well as the strategies offered by the GP opinion to address them, are described in Table 3.

**Table 3:** Fidelity of each AD session, across the four elements of adherence; challenges raised by GPs in the eight AD sessions, and strategies offered by the GP opinion to address them

					**Adherence *n*/*N* (%)**				
**Adherence area**	Session 1	Session 2	Session 3	Session 4		Session 5	Session 6	Session 7	Session 8
Duration	1/1 (100)	1/1 (100)	0/1 (-)	0/1 (-)		1/1 (100)	0/1 (-)	1/1 (100)	1/1 (100)
Coverage	4/4 (100)	4/4 (100)	4/4 (100)	4/4 (100)		4/4 (100)	4/4 (100)	4/4 (100)	4/4 (100)
Frequency	2/3 (67)	2/3 (67)	1/3 (33)	3/3 (100)		2/3 (67)	1/3 (33)	2/3 (67)	3/3 (100)
Content	7/7 (100)	6/7 (86)	7/7 (100)	7/7 (100)		6/7 (86)	7/7 (100)	7/7 (100)	7/7 (100)
Total	14/15 (93)	13/15 (87)	12/15 (80)	14/15 (93)		13/15 (87)	12/15 (80)	14/15 (93)	15/15 (100)
**Issues GP raised** **(Clinical guideline category)**		**Strategies proposed by opinion leader GPs**							
**1—What tools can assist a GP in diagnosing and assessing the severity of a MHC**									
How to diagnose a patient with MHC? How to quantify the severity of a patient’s MHC?		• Encouraged the use of diagnostic tools outlined in the guideline as they have strong evidence recommendations, compared to other questionnaires such as K10, which is relatively less specific • Encouraged to use specific diagnostic tools and questionnaires for different mental health conditions (MHC), such as depression (PHQ-9), anxiety (DASS), post-traumatic stress disorder (PCL-C) • Explained that using diagnostic tools and questionnaires increases the accuracy of diagnosis, as it is difficult to diagnose MHCs compared to ruling out MHCs in the GP setting • Use diagnostic tools in assess and monitor the progression of patients’ MHC over time • Use of diagnostic tools to convey the diagnosis of a MHC to patients, especially when they are hesitant to accept such diagnosis							
GP disagrees with some patients’ diagnosis of MHC		• Refer to independent medical practice for assessment • Still introduce management and discuss with patients regarding other factors causing their symptoms • Manage patient emotions, explore if there are underlying reasons for patients to feel/act in this specific way							
**3—Has the MHC arisen as a result of work**									
Assessing whether a MHC is work-related		• Consider the temporal relationship when assessing a MHC (i.e. the relationship between work factors and the development of the MHC)							
Diagnosing if MHC is work related when: Work injury is related to dynamics of work environment (e.g. workplace bullying) rather than the nature of work		• OP acknowledged that it is difficult to deal with and diagnose the MHC relating to workplace environment • Workplace culture has a big impact of the MHC of employees • Many patients present with MHC because of mistreatment at workplace • May chose to contact workplace with patient consent. Ask for a mediator to raise concerns on behalf of the patient							
**4—What should GP consider when conveying a diagnosis of mental health condition**									
Some patients are reluctant to accept MHC diagnosis Raised concerns of diagnosing patients with MHC, as it will be in the medical record and have other unforeseeable implications		• Avoid stigma when explaining and educating the patient regarding the condition and future management • Build therapeutic alliance • Provide information for them to read individually • Arrange next appointment and offer alternatives • Informing the patients of their conditions by explaining the diagnostic criteria and questionnaires							
**5—How can the condition be managed effectively to improve personal recovery or return to work?**									
Delays of referring to psychologist and psychiatrists, especially those who accept work cover		• Agreed that it is very difficult, especially in rural areas. Acknowledged that a long wait time is bad for patient’s health • Try to establish personal network with psychologists and psychiatrists • Keep detailed notes and arrange regular appointments with patients • There are once-off consultations at some hospitals (e.g. Bendigo Hospital) • Recommended virtual organisations such as *Dokotela* • Consider sending patient to emergency if required							
What can GPs do if patients are worried about re-injury after return to work?		• Involve independent medical examiner if second opinion is required • Educate patient about the benefits of return to work • Educate patient about the medical system and Workers’ Compensation system • Write detailed physical restrictions at work when talking about alternative duties							
**10—What can a GP do for a patient whose mental health condition is not improving**									
How to work with patients who are resistant to management		• Book longer appointments, and make sure to book follow-up appointments • Educate and explain to patients about their MHC • Involve family when necessary • Keep detailed notes about each appointment • It is important for GPs themselves to maintain a positive mindset and understand that they cannot have everything under control all the time • Refer to another GP when necessary							
**Other: Workers’ Compensation related**									
What to do when patient is hesitant to apply for Work Cover, e.g. due to concerns such as being discriminated against at workplace, or difficulties finding the next job due to records of Work Cover application		• Close communication with the workplace, with patients’ consent • Determine the state of the patient, ensure patient condition is stable, not at risk of self-harm, etc • Inform workplace and see if things can be resolved prior to submission of Work Cover claims • Keep detailed notes for each appointment • Provide patients with options, and offer to discuss in later sessions							
Difficulty managing documentation for sensitive patient information (e.g. drinking abuse) when seeking Workers’ Compensation		• Think carefully regarding what to put into files • Consider confidentiality when communicating with workplace							
Difficulty managing patients with chronic/multifactorial MHC when applying for Workers’ Compensation		• Workers’ Compensation has clear exclusion causes, which can be used to rule out • Monitor patient’s symptoms and disease progression as decline in claims may deteriorate the MHC of patients							

The average length of the eight AD sessions was 47 min, with five of the eight sessions, falling within the expected range of 50 ± 10 min. Of the remaining AD sessions, two sessions had a duration less than 40 min and one session had a duration greater than 60 min.

As per the planned design, a GP opinion leader and an academic detailer were present and co-delivered each of the eight AD sessions. All eight AD sessions had all participating GPs from the practice in attendance.

All sessions involved a discussion of the challenges GPs faced in clinical practice, opened by either the academic detailer or opinion leader, and the discussion of one case study (see Additional File 3 for case studies). In six of the eight sessions, GPs chose to discuss the case study of the patient with a secondary mental health condition. The remaining two sessions discussed the case study of the patient with a primary mental health condition.

A broad range of clinical questions from the mental health guidelines were addressed across the eight AD sessions. Additional File 4 displays the frequency with which clinical questions were discussed in the AD sessions.

A content analysis of the content of the recorded sessions revealed a number of challenges GPs face and provided insight on strategies proposed by the opinion leaders to address GP challenges. A significant challenge raised by multiple GPs was the difficulty patient’s faced accessing specialist care, such as psychologists, psychiatrists, and pain specialists. Opinion leaders acknowledged this and the long wait time for patients and offered strategies such as building a personal network of specialists and creating a safety net for patients during the long waiting period with regular appointments.

Questions regarding when and how to access Workers’ Compensation were also raised frequently by GPs during AD sessions. There was a great amount of uncertainty amongst GPs regarding Workers’ Compensation Claims when managing work-related mental health conditions. However, the ‘Clinical guideline for the diagnosis and management of work-related mental health conditions in general practice’ does not provide advice to GPs with regard to engaging with or navigating workers’ compensation claims processes across Australia.

In one instance, a GP admitted that in most cases when dealing with patients with work-related mental health conditions, they would simply refer their patients with mental health concerns to psychologists. However, after considering clinical question 9 ‘Why isn’t the patient improving?’, the GP felt empowered to play a more active role in their patients’ care.

‘Having the checklist of things here is really helpful to run through and help patients improve.’ [GP participant].

Whilst the AD plan specified that participating GPs should be encouraged to create strategies for implementing the guidelines, the opinion leaders mostly offered strategies to the challenges that GPs raised, rather than the GPs coming up with strategies themselves. Most strategies suggested were evidence-based recommendations in line with the clinical guidelines, including the use of diagnostic tools, building a therapeutic alliance, assessing if the mental health condition is work-related and facilitating a phased return to work for patients. In addition to the guidelines, other strategies that opinion leaders suggested included referring patients to an Independent Office of Review, facilitating a phased return to work for patients, and suggesting that GPs put the guideline on their desks for easy access.

The AD session focused on delivering three key messages: (i) treat the person according to their situation; (ii) offer care that is within your scope of practice and use your network to provide collaborative care; and (iii) good work is good for recovery (going to work is part of the recovery).

Additional File 5 provides a summary of how the three key messages were delivered across AD sessions.

## Discussion

We were able to demonstrate high fidelity (89.2% on average) in the delivery of the AD component of the IMPRovE intervention using the Conceptual Framework for Implementation Fidelity, as proposed by Carroll et al. [7] and modified by Hasson [10] (see Fig. 1).

High fidelity has been characterised as 80–100% adherence, with less than 50% adherence representing ‘low fidelity’ of intervention delivery [20]. Interventions with a detailed protocol for delivery are more likely to be implemented with higher fidelity compared to ambiguously described interventions [10]. Our results likely reflect the effort made to train and equip the facilitators for effective intervention delivery, as well as provision of a clear agenda and facilitation guide for the academic detailing sessions.

Our high fidelity scores were due to adequate intervention duration, the guideline being discussed frequently, as well as a good coverage of the content delivered throughout the AD sessions. There were however a number of departures from the original academic detailing protocol, and some insights of why these departures occurred are presented below.

The sessions were delivered within the intended duration of 50 min ± 10 min. However, it should be noted that in the sample, three of the eight sessions had durations outside the intended range. For sessions with shorter durations, GPs being better versed and confident in guideline recommendations and thereby requiring less time to go over points may have impacted session duration. For sessions with longer durations, these factors may include more intensive discussions about challenges faced by the GPs’ practices, GPs having more questions about clinical guideline recommendations requiring more time for the opinion leader GP to answer, or opinion leaders being unprepared, which may have resulted in more administrative activities at the start of sessions.

Throughout the sample of eight AD sessions, more GPs chose the case study for managing a patient with a secondary mental condition. This suggests GPs face a greater challenge or interest in the management of secondary mental health conditions. This may imply that, when educating GPs about work-related mental health conditions, more resources should be allocated towards discussing secondary mental health conditions to assist GPs with diagnosis and management of these kinds of mental health conditions. [21] It should also be noted that clinical question 8 ‘managing comorbid mental health conditions and substance misuse and addictive disorders’ was rarely referred to during the eight sessions. This may suggest that across all AD sessions, clinical question 8 was not addressed adequately. The lack of referral to the recommendations for this question suggests that additional resources may be need to be provided through other means, e.g. through a community of practice.

This study holds the view that interventions should be tailored for participants in different settings, whilst not so flexible that it compromises validity [2]. For instance, one GP may face a broad range of challenges, as such, a broad range of clinical questions will be addressed; whereas for another GP, they may face fewer challenges, therefore, fewer clinical questions will be addressed. Using these examples, the first session may score higher for frequency and therefore fidelity, whilst the second AD session (delivered in a targeted manner) will receive a lower fidelity score. Therefore, successful intervention delivery only requires that its ‘essential’ components be strictly delivered [21], which in the case of our AD sessions, involved delivery of the three key messages and addressing GP challenges.

AD was one component in a multifaceted behavioural intervention. There are several interacting factors that may have influenced delivery of the AD session, apart from what could be gleaned from a review of the sessions. These factors will be understood in greater depth and explained through a whole of trial process evaluation. This process evaluation for the study will be published separately.

Our approach to fidelity assessment had many advantages. Firstly our data collection, through recording the zoom sessions, was unobtrusive and meant that the researchers did not influence the results. Secondly, content analysis presents a systematic procedure that is easily replicable. Thirdly, it is a highly flexible method requiring little resources to conduct. Finally, this study reinforced the value of utilising a mixed method study design to assess complex behavioural intervention fidelity, allowing us to draw on results from both quantitative and qualitative datasets to inform our understanding of AD fidelity. This research method is not common practice for assessing behavioural intervention fidelity [22, 23], but is gaining more widespread use [15].

There were however several limitations. Firstly content analysis can be reductive and subjective; however, this was minimised with three researchers assessing fidelity. Secondly, the sample size of eight AD sessions was small. However, we coded the recommended minimum coding of 20–40% of sessions for fidelity studies chosen from a random and stratified sample (by academic detailer and by State) across the delivery of an intervention [24], to adequately represent the total 35 AD sessions. Thirdly, the scoring criteria used to evaluate the coverage, frequency and content component of the fidelity assessments did not quantify the extent different clinical questions in the guidelines were delivered. Finally, this study addressed only the ‘adherence’ component in the Modified Conceptual Framework for Implementation Fidelity [7, 10], but provided no insight into how effective the delivery of this intervention was in ultimately achieving behaviour change in GPs. These will be addressed in a future paper reporting the process evaluation of the IMPRovE trial.

As there is no consistent approach for assessing fidelity of multifaceted behavioural interventions, future research could focus on creating a consistent, validated method for assessing the fidelity of behavioural interventions in general and of AD sessions in particular. Recent modifications have been suggested for the Modified Conceptual Framework for Implementation Fidelity, such as accounting for adaptations brought by implementers or users to how an intervention was originally designed [25]. Therefore, nesting fidelity assessment within a broader mixed method evaluation framework could lend to a more comprehensive understanding of the balance between fidelity and adaptation and its impact of the intervention on outcomes. Additionally, future research could look to determine the relationship between a comprehensive fidelity assessment including adherence and moderating factors, and clinician behaviour change. For example, Swindle and colleagues [6] proposed a classification approach for adopter behaviour that considers adopter attitude and influence towards the intervention. Developing a consistent protocol for fidelity assessment incorporating these adopter factors would allow further understanding on how the implementers effort to tailor or adapt the intervention to the adopter behaviour, may impact on final trial outcomes, and provide insight for improvements in best practice. Finally, developing a more sophisticated coding scheme that quantifies the extent to which GPs’ challenges were addressed and different elements were delivered could help refine AD and other interventions components and delivery modes, towards better behaviour change outcomes.

## Conclusions

Although the use of the framework to assess intervention fidelity has not fully been established, in this study [8, 26–28], we found the Modified Conceptual Framework for Implementation Fidelity [7, 10] presents a valid method to assessing implementation fidelity of complex behavioural interventions in general practice settings. Our results provide support for using a mixed method approach in future studies that assess the implementation fidelity of complex behavioural interventions in general practice. This is particularly insightful as fidelity remains underreported in implementation studies [29].

Results from this study should give confidence that the AD portion of the IMPRovE trial intervention was delivered with high fidelity across all AD sessions. As such, there is greater confidence that the IMPRovE trial outcomes reported in the future will be the result of the AD sessions being delivered according to the protocol. High fidelity delivery also allows for a more accurate evaluation of the efficacy of this intervention on trial outcomes.

### Supplementary Information

**Additional file 1. **The IMPRovE trial.

**Additional file 2. **Assessment sheet used for the fidelity coding.

**Additional file 3. **The case studies of the primary mental health patient or secondary mental health patient seen.

**Additional file 4. **Frequency with which clinical questions were discussed in the AD sessions.

**Additional file 5. **Key messages delivered in AD sessions.

## Data Availability

Trial data can be obtained upon request from the corresponding author.

## Abbreviations

AD: Academic detailing
GP: General practitioner
IMPRovE: Implementing work-related Mental health guidelines in general PRacticE
