# Supplementary material for: Assessing the fidelity of a behavioural intervention involving academic detailing in general practice: a sub-study of the ‘Implementing work-related Mental health guidelines in general PRacticE’ (IMPRovE) trial
Source: Implement Sci Commun. 2023 Nov 29;4:154. doi: 10.1186/s43058-023-00531-2 (PMC10687810; doi:10.1186/s43058-023-00531-2)
Supplement: Supplementary file 1 — Additional file 1. The IMPRovE trial. [file 43058_2023_531_MOESM1_ESM.docx]

**Additional File 1: The IMPRovE trial**

Work-related mental health conditions affect approximately 210,000 people each year, and are the fastest growing and most expensive compensable condition in Australia [1]. General Practitioners (GPs) see 97% of injured workers [2], and are vital to supporting these patients, but face many challenges in providing care to this patient group. The 'Clinical guideline for the diagnosis and management of Work-Related Mental Health conditions in general practice' (work-related mental health guideline) [3] was developed in response. It addresses the top ten clinical questions that GPs identified as major challenges (see two-page guideline summary for GPs in Appendix A).

The 'Implementing work-related Mental health guidelines in general PRacticE' (IMPRovE) trial was a pragmatic parallel cluster randomised controlled trial of a complex intervention designed to improve GP’s adherence to the work-related mental health guideline [4].

Participating GPs were those who felt they could recruit the required number (7-24 during the course of the trial) of eligible patients (> 18 years, employed, with a confirmed or suspected work-related mental health condition, receiving care from that GP).

The trial involved three intervention components: 1) receipt of AD highlighting the evidence-based guideline recommendations and their ability to help GPs overcome the challenges they face dealing with the diagnosis and management of work related health mental health conditions in clinical practice, 2) enrolment and engagement in an online community of practice to provide ongoing support and peer networking in relation to implementation of the guideline recommendations, and 3) receipt of guideline resources.

**Description of the IMPRovE trial AD sessions**

The IMPRovE trial AD sessions were educational outreach sessions (similar to a tutoring program) co-delivered by a GP-opinion leader and an experienced academic detailer, to intervention GPs via the Zoom online platform. A facilitation manual was developed to support the delivery of the sessions which were planned to last up to 60-minutes. The AD session focused on delivering three key messages:

1. Treat the person according to their situation;
2. Offer care that is within your scope of practice and use your network to provide collaborative care; and
3. Good work is good for recovery (going to work is part of the recovery).

During the session the GP-opinion leader was also required to: (1) identify aspects of care the GP-participants found most difficult, and in response (2) discuss one of two case studies that illustrated the application of the guideline recommendations to that challenging area of care.

Both the GP opinion leaders and the academic detailers that facilitated the sessions, underwent extensive training prior to conducting the AD sessions, involving undertaking five 30-minute long modules provided by the NPS that covered aspects of providing academic detailing to clinicians, familiarisation with the facilitator manuals and practicing the delivery of the AD sessions. Additionally, a fidelity assessment report on the first five AD sessions was conducted before further sessions were delivered, and recommendations for improvement were fed back to the detailing team via email.

Figure 1 outlines the agenda for the AD sessions delivered. GP opinion leaders were asked to lead the discussion while the academic detailers ensured that the structure of the session followed the agenda.


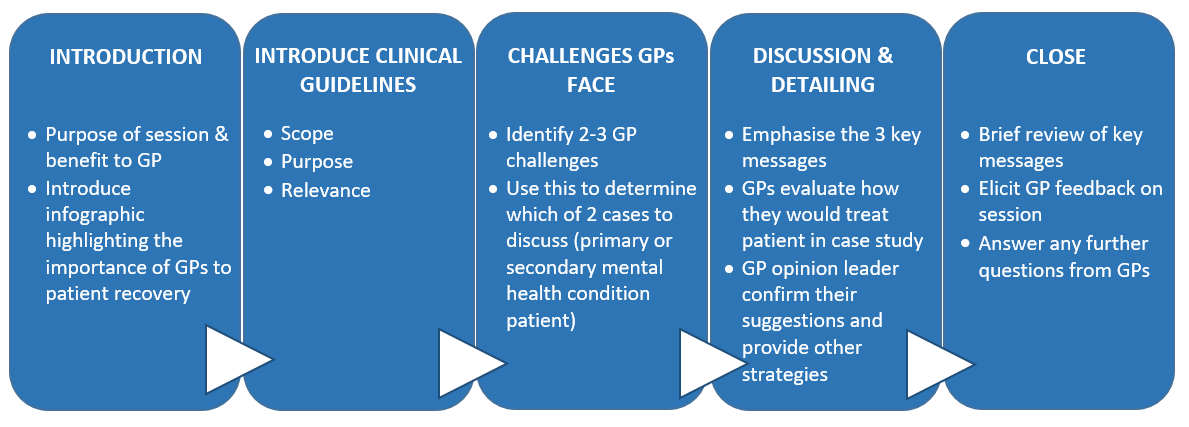


**Figure 1: Academic detailing session agenda**

References:

1. Australia, S.W., Work-related mental disorders profile 2015, S.W. Australia, Editor. 2015: Canberra.

2. Dembe, A.E., et al., Inpatient hospital care for work-related injuries and illnesses. Am J Ind Med, 2003. 44(4): p. 331-42.

3. Mazza, D., B. Brijnath, and S. Chakraborty, Clinical guideline for the diagnosis and management of work-related mental health condition in general practice–Public Consultation Submission Summary. 2019, Melbourne: Monash University.

4. Mazza, D., et al., Implementing work-related Mental health guidelines in general PRacticE (IMPRovE): a protocol for a hybrid III parallel cluster randomised controlled trial. Implementation Science, 2021. 16(1): p. 77.

5. Flodgren, G., et al., Local opinion leaders: effects on professional practice and healthcare outcomes. Cochrane Database Syst Rev, 2019. 6(6): p. Cd000125.

**
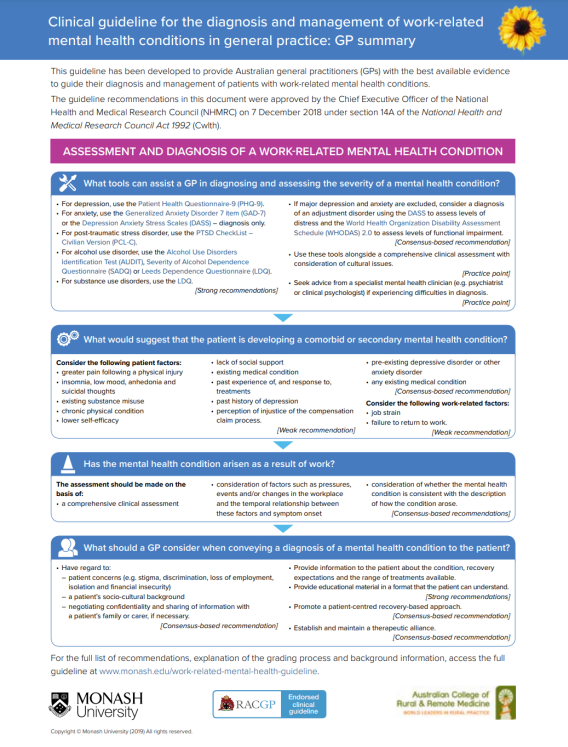
Appendix A: 2-page summary of the ‘Clinical Guidelines for the diagnosis and management of mental health conditions in general practice’**

**
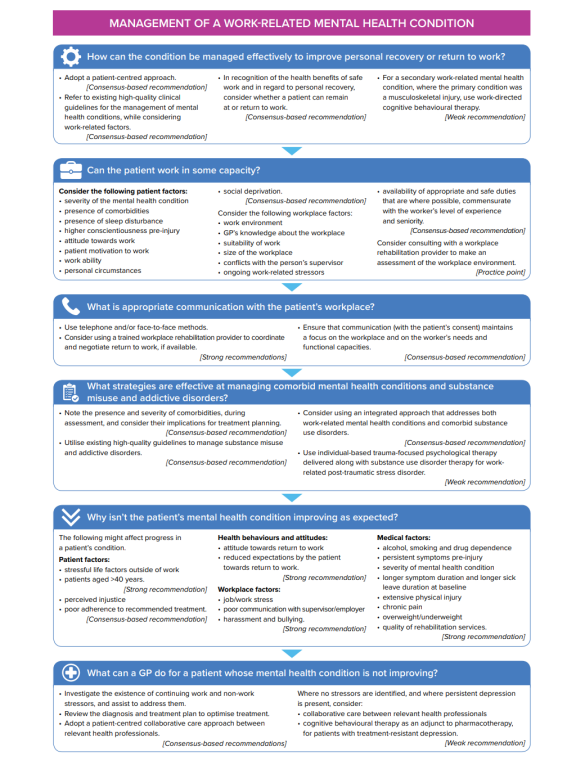
**
