# Supplementary figures and images for: Assessing the fidelity of a behavioural intervention involving academic detailing in general practice: a sub-study of the ‘Implementing work-related Mental health guidelines in general PRacticE’ (IMPRovE) trial
Source: Implement Sci Commun. 2023 Nov 29;4:154. doi: 10.1186/s43058-023-00531-2 (PMC10687810; doi:10.1186/s43058-023-00531-2)

**
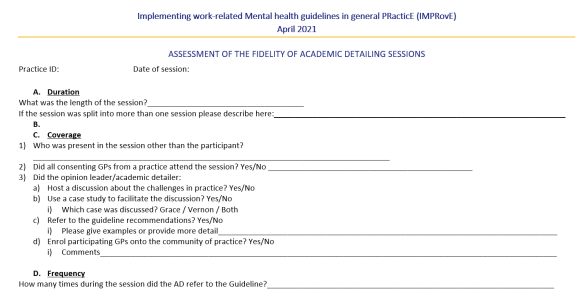
Additional File 2: Assessment sheet used for the fidelity coding**

**

**

Supplement: Supplementary file 2 — Additional file 2. Assessment sheet used for the fidelity coding. [file 43058_2023_531_MOESM2_ESM.docx]
