# Supplementary material for: Assessing the fidelity of a behavioural intervention involving academic detailing in general practice: a sub-study of the ‘Implementing work-related Mental health guidelines in general PRacticE’ (IMPRovE) trial
Source: Implement Sci Commun. 2023 Nov 29;4:154. doi: 10.1186/s43058-023-00531-2 (PMC10687810; doi:10.1186/s43058-023-00531-2)

**Additional File 3: The case studies of the primary mental health patient or secondary mental health patient seen**

Case study of patient with a secondary mental health condition


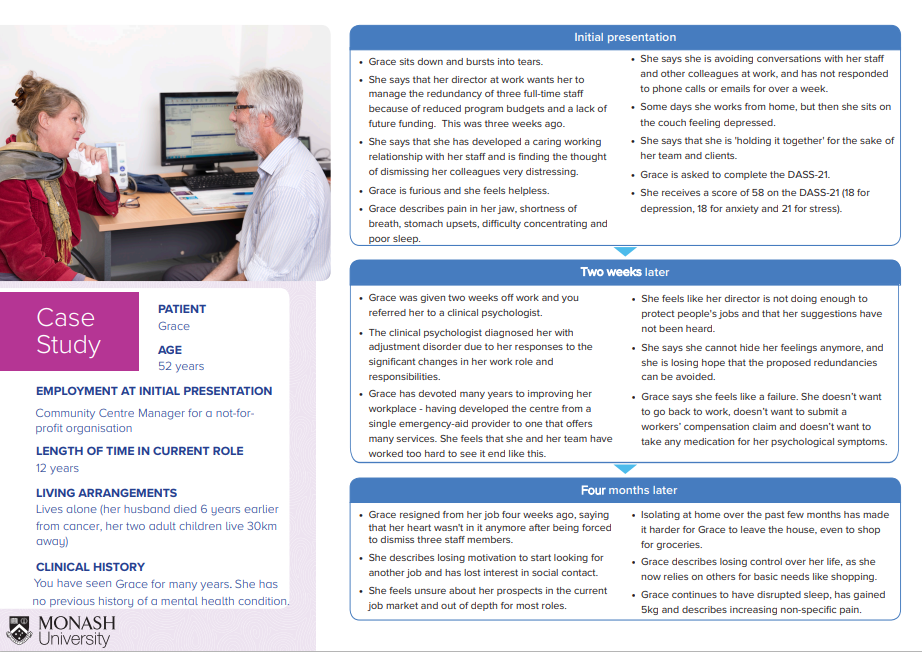


Case study of a patient with a primary mental health condition


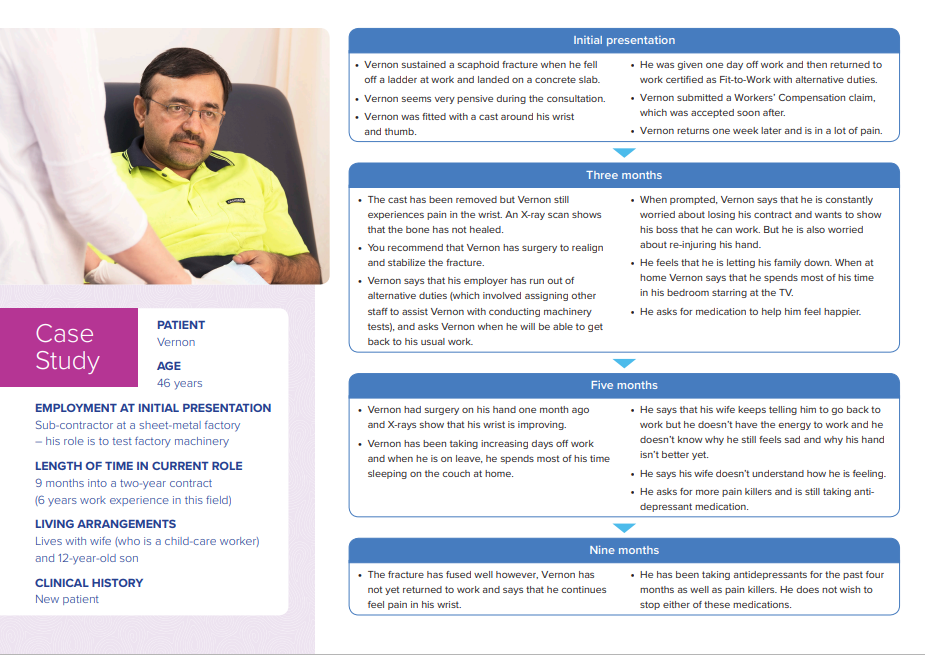

Supplement: Supplementary file 3 — Additional file 3. The case studies of the primary mental health patient or secondary mental health patient seen. [file 43058_2023_531_MOESM3_ESM.docx]
