# Supplementary material for: Assessing the fidelity of a behavioural intervention involving academic detailing in general practice: a sub-study of the ‘Implementing work-related Mental health guidelines in general PRacticE’ (IMPRovE) trial
Source: Implement Sci Commun. 2023 Nov 29;4:154. doi: 10.1186/s43058-023-00531-2 (PMC10687810; doi:10.1186/s43058-023-00531-2)
Supplement: Supplementary file 4 — Additional file 4. Frequency with which clinical questions were discussed in the AD sessions. [file 43058_2023_531_MOESM4_ESM.docx]

**Additional File 4: Frequency with which clinical questions were discussed in the AD sessions**

Overall, four of the ten clinical questions were addressed in all eight sessions, four clinical questions were addressed in seven sessions and one clinical question was addressed in six sessions. Clinical question 8 was only addressed three times across all sessions, and question 1 was addressed with the highest frequency overall. Table 1 displays the frequency that the clinical questions of the guideline were referred to across the eight academic detailing sessions.

**Table 1 - Frequency that the clinical questions of the guideline were referred to across the eight academic detailing sessions**

| **Clinical question number** | **Guideline question** | **Frequency discussed overall** | **Number of AD sessions where the question was discussed** |
| --- | --- | --- | --- |
| 1 | What tools can assist a GP in diagnosing and assessing the severity of a mental health condition? | 14 | 8 |
| 7 | What is appropriate communication with the patient's workplace? | 13 | 8 |
| 3 | Has the mental health condition arisen as a result of work? | 10 | 8 |
| 9 | Why isn't the patient's mental health condition improving as expected? | 11 | 8 |
| 4 | What should a GP consider when conveying a diagnosis of a mental health condition to the patient? | 9 | 7 |
| 2 | What would suggest that the patient is developing a comorbid or secondary mental health condition? | 8 | 7 |
| 6 | Can the patient work in some capacity? | 9 | 7 |
| 10 | What can a GP do for a patient whose mental health condition is not improving? | 9 | 7 |
| 5 | How can the condition be managed effectively to improve personal recovery or return to work? | 8 | 6 |
| 8 | What strategies are effective at managing comorbid mental health conditions and substance misuse and addictive disorders? | 4 | 3 |
