# Supplementary material for: Assessing the fidelity of a behavioural intervention involving academic detailing in general practice: a sub-study of the ‘Implementing work-related Mental health guidelines in general PRacticE’ (IMPRovE) trial
Source: Implement Sci Commun. 2023 Nov 29;4:154. doi: 10.1186/s43058-023-00531-2 (PMC10687810; doi:10.1186/s43058-023-00531-2)

**Additional File 4: Key messages delivered in AD sessions**

In all AD sessions, Key message 1 and 2, ‘Treat the person according to their situation’ and ‘Offer care that is within your scope of practice and use your network to provide collaborative care’, respectively, were delivered as intended. Table 1 provides a summary of the three key messages and the ways in which they were delivered across AD sessions.

**Table 1 - The three key messages delivered in the AD sessions**

| **Key message** | **Approach to delivering each key messages within AD sessions** |
| --- | --- |
| 1. Treat the person according to their situation | • Using the diagnostic tools to diagnose, assess the patient's mental health severity and as a marker for the progression of their mental health condition |
|  |  |
|  | • Establishing a therapeutic alliance with patients: |
|  | ➤ being nonjudgmental and understanding that patients can be |
|  | ➤ devising acceptable and accessible treatment options that the patient will follow |
|  |  |
|  | • Setting up a recovery-oriented approach with a realistic yet optimistic return to work date in mind |
|  | ➤ discussion of the recovery expectations for the patient |
|  | ➤ provide patients education materials to help them understand the mental health condition |
|  |  |
|  | • Set a patient-centred approach, whether they would like to involve any other individuals (i.e., partner) in future consultations |
|  | • Investigate the existence of continuing work-related and non-work-related stressor that contribute to delayed patient recovery and assist to address them |
|  | • Regularly review the diagnosis and treatment plan to ensure the patient is receiving optimal care |
| 2. Offer care that is within your scope of practice and use your network to provide collaborative care | • Engage in collaborative care with other health providers including physiotherapists, occupational therapists, rehab providers or psychologists so the patient can receive optimal care by another health professional with expertise in that area |
|  | • Engage in case conferences that include the employer, patient, or other health-care providers |
| 3. Good work is good for recovery | • Emphasized the value of work for recovery to the patient |
|  | • Facilitate a phased return to work for patients if the workplace provides a safe environment for the patient |
|  | • Assess the patient's beliefs and attitude on their mental health condition, physical injury, treatment options and toward the workplace |
|  | • Consider alternative duties for the patient where they return to work |
|  | • Emphasise the employer’s role in establishing a safe work environment |

Key message 3, ‘Good work is good for recovery’ was not delivered with the meaning intended in session 5. It was interpreted as “if GPs do a good job of treating patients, then that is good for patient recovery”. The intended interpretation was that returning to work is part of the patient’s recovery.

**Other content**

One aspect of the academic facilitation manual was not delivered in any AD sessions. This involved making reference to the following two tools: (1) The Work Environment Subscales of the Work Health Check [1], and (2) the Workplace Stressors Assessment Questionnaire [2].

In all eight AD sessions, the importance of GPs to the patient recovery were described using the handout in Appendix A

References

1. Gadinger, M.C., et al., The Work-Health-Check (WHC): a brief new tool for assessing psychosocial stress in the workplace. Work, 2012. 43(3): p. 345-60.

2. Mahmood, M.H., et al., Development and testing of the Workplace Stressors Assessment Questionnaire. J Occup Environ Med, 2010. 52(12): p. 1192-200.

**Appendix A - Handout for the importance of GPs to patient recovery**


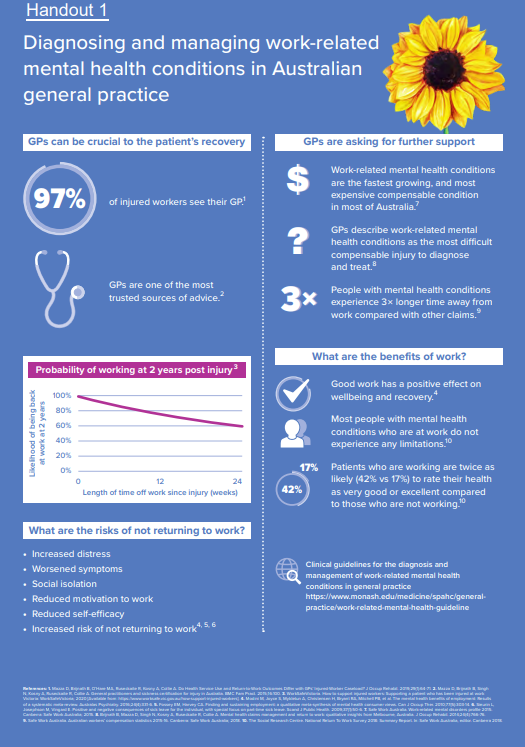

Supplement: Supplementary file 5 — Additional file 5. Key messages delivered in AD sessions. [file 43058_2023_531_MOESM5_ESM.docx]
